# Alterations in Hippocampal Oxidative Stress, Expression of AMPA Receptor GluR2 Subunit and Associated Spatial Memory Loss by *Bacopa monnieri* Extract (CDRI-08) in Streptozotocin-Induced Diabetes Mellitus Type 2 Mice

**DOI:** 10.1371/journal.pone.0131862

**Published:** 2015-07-10

**Authors:** Surya P. Pandey, Hemant K. Singh, S. Prasad

**Affiliations:** 1 Biochemistry & Molecular Biology Lab, Centre of Advanced Study in Zoology, Banaras Hindu University, Varanasi, 221005, Uttar Pradesh, India; 2 Lumen Research Foundation, Ashok Nagar, Chennai, 600083, Tamilnadu, India; University of Lancaster, UNITED KINGDOM

## Abstract

*Bacopa monnieri* extract has been implicated in the recovery of memory impairments due to various neurological disorders in animal models and humans. However, the precise molecular mechanism of the role of CDRI-08, a well characterized fraction of *Bacopa monnieri* extract, in recovery of the diabetes mellitus-induced memory impairments is not known. Here, we demonstrate that DM2 mice treated orally with lower dose of CDRI-08 (50- or 100 mg/kg BW) is able to significantly enhance spatial memory in STZ-DM2 mice and this is correlated with a significant decline in oxidative stress and up regulation of the AMPA receptor GluR2 subunit gene expression in the hippocampus. Treatment of DM2 mice with its higher dose (150 mg/kg BW or above) shows anti-diabetic effect in addition to its ability to recover the spatial memory impairment by reversing the DM2-induced elevated oxidative stress and decreased GluR2 subunit expression near to their values in normal and CDRI-08 treated control mice. Our results provide evidences towards molecular basis of the memory enhancing and anti diabetic role of the *Bacopa monnieri* extract in STZ-induced DM2 mice, which may have therapeutic implications.

## Introduction

Diabetes mellitus (DM) is a metabolic disorder characterized by abnormally increased blood glucose level and is synonymously called as hyperglycemia. DM has been clinically classified as insulin dependent diabetes mellitus (IDDM or type 1 DM/DM1) or non insulin-dependent diabetes mellitus (NIDDM or type 2 DM/DM2) based on decline in blood insulin level due to its poor secretion from β cells of islets of Langerhans in the pancreas or increased resistance of insulin leading to its poor targeting action on glucose metabolism, respectively followed by disturbances in the fat and protein metabolism [1]. Prolonged and untreated hyperglycemia due either to type-1or type-2 DM has been reported to cause several pathological complications such as retinopathy [2], nephropathy, neuropathy and microvascular complications in cerebral as well as cardiac arteries [3–5]. Numerous studies on untreated DM human patient have shown deposition of interneuronal amyloid plaques in the brain which thereby leads to development of neuropathological symptoms of Alzheimer’ disease (AD) [6]. DM has been reported to be associated with its negative impacts on the function of central nervous system [6,7]. Recent evidences from human DM patients suggest that the insulin resistance leading to type-2 DM, in particular, causes impairments in the visual retention, verbal memory, working memory, immediate recall, executive function, information processing speed, verbal fluency, attention, and depression, which ultimately lead to cognitive dysfunction [8–10]. Evidence also suggests that successful management of DM does not result into complete reversal of the cognitive impairments [11,12]. The precise mechanism underlying the DM-induced cognitive dysfunction is not fully understood. However, number of studies from human DM2 patients and rodent DM2 model reveal that excessive oxidative stress leads to neurodegeneration as a consequence of impaired glucose metabolism and excessive operation of polyol-sorbitol pathway in neurons [13,14], altered excitatory glutamatergic synaptic transmission, early long term potentiation (eLTP) and synaptic plasticity, which altogether result into cognitive impairments [14,15].

Development of eLTP in the hippocampus, the gateway of learning, memory and cognition, requires sequential activation of the two major ionotropic glutamate receptors called α-amino-3-hydroxy-5-methyl-4-isoxazole propionate (AMPA) and N-methyl-D-aspartate (NMDA) receptors at glutamatergic synapse. Following the presynaptic neuronal signaling, glutamate is released in the synaptic cleft and activates the AMPA receptors at the post synaptic density. Glutamate activated AMPA receptors cause depolarization of the postsynaptic neuronal membrane, which in turn, acts as a signal for opening of the NMDA receptors by removal of its Mg^2+^ block to achieve post synaptic potential (PSP) and allows diffusion of extracellular Ca^2+^ and Na^+^ into postsynaptic neurons. This leads to rise in the intracellular Ca^2+^ level, binding of Ca^2+^ with calmodulin (CaM) and activation of CaMKIIα, which in turn achieve Ca^2+^-dependent synaptic plasticity mediated alterations in the density and activity of both the glutamate receptor types on the post synaptic density. AMPA receptors possess heterotetrameric structure consisting of four subunits designated either as GluA1–GluA4 or GluR1–GluR4 or GluRA- GluRD) [16–19]. Among them, the GluR2 subunit plays important role in synaptic plasticity as it is inserted into membrane during generation of LTP or it is internalized into cytosol during LTD [18,20,21]. Based on recent studies on DM2rodent models, it has been shown that expression of AMPA and NMDA types glutamate receptors and kinetics of their binding with glutamate are abnormally altered which thereby leads to excitotoxicity in the cerebral cortex [22] and abnormal alterations in hippocampal synaptic plasticity [23].

*Bacopa monnieri*, commonly known as Brahmi, is one of the most common Indian herbs that are known for its nootropic role in array of neurological disorders and memory impairments [24]. Its alcoholic extract has been shown to possess antioxidant activity based on number of studies on various animal disease models [25–28] and used in neuroprotection [29], cognition [30], depression [31], anxiety [32], epilepsy [33], cancer [34] and inflammation related disorders [35]. Existing literatures suggest that *Bacopa* extract possesses neuroprotective effects on various memory related areas of the brain i.e. frontal cortex, hippocampus and striatum, and enhance learning and memory due to its antioxidant properties [25]. The active ingredients of *Bacopa monnieri* alcoholic extract prepared from leaves or stems of the plant have been characterized. These include Bacosides A1–A3, Bacosides I–III and Bacosaponins A–G [36]. Recent studies have linked nootropic and neuroprotective role of *Bacopa* extract with its two major saponins- Bacosides A and B [29,37]. Ethanomedicine researchers have been keenly looking for the precise molecular mechanism underlying the action of its extract in recovery of the cognitive loss due to DM. The role of the CDRI-08, a fraction of *Bacopa monnieri* extract rich in Bacosides A and B, in the DM-induced alterations in oxidative stress and expression of the AMPA or NMDA receptors and thereby the synaptic plasticity is not well established.

Existing literatures suggest that diabetes mellitus leads to increase in the oxidative stress, which is associated with altered synaptic plasticity and this in turn leads to decline in memory. Therefore, in the current study, we have developed the DM2 mice model by intraperitoneal injection of STZ, validated by assessing various diabetic parameters, insulin resistance and studied effects of STZ-induced DM2 on the spatial memory, oxidative stress and expression of the AMPA receptor GluR2 subunit gene at protein and transcript levels in the hippocampus and compared with values in the normal as well as CDRI-08 controls. Further, since one of the foremost issues related to using an optimum dose of CDRI-08 for its role in above has not been well established, the current study includes analysis of effects of its different doses on the level of lipid peroxidation as one of the markers of oxidative stress and expression of the AMPA receptor GluR2subunit as a marker of synaptic plasticity in the hippocampus of the streptozotocin (STZ)-induced DM2 mice as compared to their values in the normal and CDRI-08 treated control mice.

## Materials and Methods

### Chemicals and reagents

All chemicals were of analytical and molecular biology grade. They were purchased from Sigma, USA or Merck, India. Anti GluR2 primary antibody was obtained from Antibodies Incorporated (Neuromab, UC Davis, USA) and HRP-conjugated secondary antibody against anti-mouse primary antibodies was purchased from Genie, Bangalore, India. Streptozotocin (STZ) and Tween 80 were purchased from Sigma, USA. Standardized extract of *Bacopa monnieri* called CDRI-08 (containing 55 ± 5% of Bacosides A and B) was obtained as a kind gift from Mr. S. Selvam, Lumen Research Foundation, Chennai, India). Gene specific primers for semi quantitative RT-PCR were custom synthesized by the Imperial Life Science, USA.

### Animals, induction of diabetes mellitus type II (DM2) and CDRI-08 treatment

Male pups of 1–2 day age obtained from Swiss strain albino mice maintained at 25 ± 2°C RT with 12:12 hr light and dark cycle were used in the experiment. The present study was approved by Animal Care and Use Committee (IACUC) of Banaras Hindu University and the procedure for use and handling of animals were in accordance with its guidelines. DM2 mice model was developed following the method as described earlier [38]. However, to ascertain the optimum dose of STZ to develop DM2 mice model, a pilot study on the effects of single injection of various doses of STZ (such as 50-, 75-, 100-, 125- or 150 mg/kg BW) on the blood glucose levels was separately performed, and blood glucose content and mortality of mice pups were monitored every four weeks. It was observed that 100 mg/kg BW of the STZ was the optimum dose to induce abnormal increase in blood glucose level without causing mortality in mice compared to its higher doses (125 mg/kg BW and above) (S1 Fig). Thereafter, a batch of pups (n = 60) was intraperitoneally injected with a single dose of freshly prepared streptozotocin (STZ) (100 mg/kg BW in 10 mM sodium citrate buffer, pH 4.5. The control pups group received equal volume of the vehicle (Citrate buffer) only. The pups were maintained with mother until they learnt to consume food pellet and water at *ad libitum* independently. Thereafter, they were maintained separately in standard laboratory condition as mentioned above for 12 weeks. From the 10^th^ week after STZ treatment, body weight, blood glucose content, water consumption (for evaluating the level of polydipsia) and volume of urine (to assess the condition of polyurea) were regularly determined following the procedures mentioned herein. Mice with blood glucose level >250mg/dL) were considered as diabetic and were further divided into 7 groups (n = 6–7) accordingly for their oral treatment with different doses of CDRI-08(50-, 100-, 150-, 200-, 250-, 300 mg/kg BW in 5% Tween80 medium as described earlier [30]. The control mice treated with citrate buffer till 10^th^ week were further given oral treatment of 5% Tween 80 for rest of the time and used as control for the effects of CDRI-08. Further, to assess the per se effects of CDRI-08 (CDRI-08 alone), a parallel CDRI-08 control batch of 6–7 citrate buffer treated mice (without STZ treatment) were orally given various doses of CDRI-08 as mentioned above and effects of the individual doses were analyzed in respect to parameters mentioned above. Mice of all groups were maintained in individual metabolic cages and were regularly examined for their body weight, serum insulin content, HOMA-IR (insulin resistance), blood glucose level, water consumption and urine output.

### Determination of body weight, blood glucose content, water consumption and urine output

In order to assess the impacts of STZ-induced DM2 and *Bacopa monnieri* extract on DM2 mice, the body weight of each mouse of control and treated groups was determined before and after completion of different treatments and the results were expressed as average body weight in terms of gram (g). Glucose content was measured in the blood obtained by puncturing the tail of mice of each experimental group using a Glucometer (Accu-Check Active, Roche Diagnostics, Mannheim, Germany) [39]. Water consumption/mouse/day was separately determined by determining the total volume of water consumed by all mice and then by dividing the total volume of water consumed by number of mice in each group. Total volume of water consumption was found out every 24 hrs by measuring the decrease in the initial volume of water (usually 50 ml) kept for *ad libitum* consumption at a fixed time every day. The result of water consumption was expressed as volume of water (ml)/mouse/day to denote the level of polydipsia, the level of thirst [40]. Further, in order to measure the volume of urine, mice belonging to control and experimental groups were separately housed in standard metabolic cage. Cages were equipped with metallic grill fixed at 2.0 cm above the floor of the cage. The floor was fixed in the cage in such a way that urine discharged by mice will be flowing towards a groove in one of the corners. Volume of the urine collected from each cage was collected for 24 hrs and measured using micropipette. The total urine output of each cage was divided by number of mice and the result was expressed as volume of urine (ml)/mouse/day) in order to assess the level of polyurea.

### Sandwich ELlSA of serum insulin and measurement of insulin resistance (HOMA-IR)

To analyze the impacts of STZ-induced DM2 and *Bacopa monnieri* extract (CDRI-08) on the serum Insulin level, the sandwich ELISA technique was used following manufacturers’ protocol (LDN GmbH, Germany). Briefly, 25 μl of the standard or the experimental serum samples collected separately from control and treated mice and 25 μl conjugate were added into anti insulin antibody coated microplate well, shacking mixed for 10 sec and allowed the mix to stay at RT for 30 min. Thereafter, the bound insulin-antibody complex was washed three times with washing solution at RT and the residual wash solution was discarded. Thereafter, 50μl of the enzyme complex was added to the reaction mixture and incubated for 30 min at RT. Thereafter, the well containing the reaction product was washed three times with washing solution as above and the residual solution was discarded. Finally, 50 μl of the chromogenic substrate was added to the well and reaction was allowed to continue for 15min. Reaction was stopped by adding 50 μl of stop solution to above reaction mix and the optical density of the resulting solution was measured at 450 nm using Microplate Reader (Bio-Rad, USA).

To study whether targeting function of the insulin (insulin resistance) was affected by different doses of CDRI-08, the homeostasis model assessment of insulin resistance (HOMA-IR)was measured by subjecting the values of fasting serum glucose (mg/dL) and fasting serum insulin (μIU/mL) levels in mice of corresponding experimental sets to the following formula: HOMA-IR = [fasting plasma glucose (mg/dL) X fasting plasma insulin (μIU/mL)] X (405)^−1^ as described by Matthews et al. [41]. The HOMA-IR values obtained from mice of individual experimental set are shown in tabular form in S1 Table.

### Morris-water-maze test

To assess whether the spatial memory is altered due to DM2 and CDRI-08 has any effect on the altered memory, mouse of each experimental group were individually subjected to Morris-Water-Maze test[42]. The maze consisted of a black painted circular pool (106 cm diameter X 76.2 cm height, filled with 24±2°C water such that its level is 2.0 cm above the surface of the hidden platform (6 cm X 6 cm) kept in the pool. The apparatus was kept in a dedicated animal behavior room. The pool was divided into four quadrants one of which housed the said platform. Visual cues were pasted on the wall of each quadrant. Before the actual assessment of the spatial memory, the mice were first acclimatized with behavior room and the maze for two days. They were trained to locate the platform during a training session. Thereafter, the individual mouse was given three acquisition trials (90 second/trial) per day for 15 consecutive days between 11 AM to 3 PM every day. The path of movement by each mouse was recorded by video tracking system. The escape latency (time taken) and path length traversed by each mouse for searching the hidden platform was analyzed by ANY-maze software (Microsoft version 4.84, USA) as described earlier [43].

### Isolation of hippocampus

Hippocampus was dissected following the procedure described by Wan [44]. Briefly, mice were treated with anaesthesia ether and sacrificed by cervical dislocation following the guidelines of IACUC of Banaras Hindu University. The skull bone was carefully removed by bilateral incision and the intact brain was carefully dissected out from the skull and placed into ice-cold phosphate-buffered saline (PBS), the adherent blood was removed and transferred onto a wet filter paper placed on ice and the hind brain and cerebellum were removed using surgical blade. Cortical layer of each hemisphere was laterally peeled out under microscope. Thereafter, hippocampus was exposed and removed from brain by applying pressure to the medial white matter tracts with a needle and moving another needle slightly anteriorly and laterally. The hippocampal tissues were pooled, dipped into liquid nitrogen and stored frozen at -70°C or used immediately.

### Estimation of malonaldehyde (MDA)

To measure the level of oxidative stress, MDA content in the hippocampal tissue homogenate was determined for quantitation of the lipid peroxidation by modified TBARS (Thiobarbituric acid-reacting substance) assay method [45]. Briefly, a 10% homogenate of the hippocampus was prepared in 0.01 M Tris-Cl buffer, pH 7.4 and the homogenate was centrifuged at 15000Xg at 4°C, the supernatant was collected and incubated in a solution containing 8.1% SDS, 20% acetic acid (pH 3.5), 0.8% TBA (Thiobarbituric acid) for 1 h at 95°C. After cooling at RT, the samples were well mixed with butanol/pyridine mixture (15:1, v/v), and the aqueous phase was carefully collected by aspiration. Absorbance of the aqueous phase was found out at 532 nm, and the MDA level was calculated using standard 1A_532_value and appropriate dilution factors as described earlier [45]. The results were expressed as nmol MDA/mg protein.

### Western blot analysis

A 20% homogenate of the pooled hippocampal tissue was prepared in TEEN buffer (50 mM Tris-Cl, pH 7.4, 1mM EDTA, 1mM EGTA, 150 mM NaCl) containing 100 μg/ml PMSF and 1μg/ml protease inhibitor cocktail. The resulting homogenate was centrifuged at 5000Xg and the resulting supernatant was collected and aliquoted in small volume. The total protein content in the supernatant was estimated by Bradford method [46]. Aliquots were directly used for further experiment or stored at -70°C. The aliquoted supernatant was mixed with sample buffer (100 mM Tris-Cl, pH 6.8), 2% SDS, 2% β-mercaptoethanol, 20% glycerol and 0.2% bromophenol blue), heated on a boiling water bath for 5 min and centrifuged at 10,000Xg at 4°C for 20 min. Thereafter, the supernatant was collected. 50μg total protein was loaded onto 10% SDS-polyacrylamide gel and electrophoresis was carried out as described earlier [47]. After completion of the electrophoresis, the gel was removed and proteins from the gel were immobilized onto polyvinylidenedifluoride (PVDF) membrane by wet transfer method. Membrane was stained with Ponceau-S in order to ensure the transfer of proteins. Then, the membrane was washed with 1X phosphate buffer saline (PBS) and was blocked with 5% non-fat milk powder dissolved in 1X PBS for 4 h at RT. The membrane was incubated with anti-GluR2 (1:2500 dilutions) primary antibody overnight and washed for 5 min in PBST (PBS containing 0.1% Tween 20). After this step, the blot was incubated with anti-mouse HRP-conjugated secondary antibody (1:2500 dilutions in PBS containing 5% non-fat milk for 4 h and then washed with PBST at RT. The blot was also processed with rabbit monoclonal anti-β-Actin antibody (1:25,000 dilutions, Sigma-Aldrich, USA side by side to examine the level of β-Actin as internal marker as described above. The signal of the specific antibody-protein complex was detected on the X-ray film by enhanced chemiluminescence (ECL) method by appropriate treatment of the membrane following the manufacturers’ protocol. The signals on the X-ray film were densitometrically scanned and quantified using computer-assisted densitometry (Alpha imager 2200). Scan value of individual protein signals was normalized with the scan value of the β-Actin and the quantitation data was expressed as relative density value (RDV) for GluR2 subunit expression.

### Immunofluorescence cytochemistry

*In situ* expression of AMPAR GluR2 subunit in the hippocampal CA3 and CA1 areas in ultra thin sections was studied by immunofluorescence cytochemistry (IFC). Mouse from each group was individually anesthetized using anaesthetic ether, 50 ml of 2% paraformaldehyde (in 1XPBS medium) was transcardially perfused to fix the brain tissue. Thereafter, the whole brain was dissected out and serially immersed in 10%, 20% and 30% sucrose solutions (w/v) for 24 h each. 14μm thick transverse sections of the brain along the hippocampus were prepared using a cryotome (Thermo Scientific, USA) and placed on poly-L-lysine (cryomount)-coated glass slides and stored in deep freezer till further use. The cryosections were incubated at 37°C for one hour till water was evaporated and the dried sections were successively washed three times (5 min each) in 1XPBS buffer (137 mM NaCl, 2.7 mM KCl, 4.3 mM Na_2_HPO4, 4.0 mM KH_2_PO4, pH 7.4) to remove the cryomount. Then, the sections were permeabilized with 1% TritonX-100 in 1X PBS buffer for 40 minutes followed by washing with 1X PBS buffer for removal of excess of Triton X-100. Thereafter, sections were treated with 10% normal goat serum for 1hr to block the nonspecific proteins. Sections were then incubated with appropriately diluted anti GluR2 primary antibody (1:250in 3% BSA-1X PBS medium) in a moist chamber at 4°C overnight. Next day, the sections were brought to room temperature and washed thrice with 1X PBS buffer for 5 minutes, and incubated with FITC-labelled anti mouse anti IgG secondary antibody raised in goat at a dilution of 1:250in 3% BSA-1X PBS buffer for 120 minutes at RT in a dark chamber. The sections were finally subjected to three successive wash with 1X PBS buffer and mounted in aqueous mounting medium (Hardset with DAPI). A negative control was included by omitting the primary antibody. Photomicrographs of GluR2 specific signals and nuclear stains were captured with fluorescence microscope (Leica DM2000) at 40X magnification. The immunofluorescence signal for GluR2 expression of was analysed by Image J software (NIH, USA) Area integrated density measurement tool.

### Total RNA isolation

Total RNA was isolated from the pooled hippocampal tissue using TRI reagent (Sigma, USA) following the supplier’s manual. The aqueous phase was collected, mixed with equal volume (v/v) of isopropanol and precipitated at -70°C. The pellet containing RNA was collected, washed with ice-cold 70% ethanol and dissolved in DEPC-treated water. The total RNA was mixed with DNase-I (DNAfree, Ambion) according to the manufacturer's guidelines to remove any DNA contaminants. RNA content in the DNA-free RNA preparation was determined by measuring the A_260_ value. The integrity of the RNA sample was examined by 1% formaldehyde-agarose gel electrophoresis [48].

### Semi quantitative reverse transcriptase-polymerase chain reaction (RT-PCR)

The total RNA was used as template for the synthesis of GluR2 and β-Actin cDNA using random hexamer primers and specific primers for GluR2 (F- CAGTGCATTTCGGGTAGG, R-TTGGTGACTGCGAAACTG) and β-actin (F- ATCGTGGGCCGCTCTAGGCACC, R- CTCTTTGATGTCACGATTTC), respectively. The RT-PCR was carried out in Thermal Cycler (G-Strom, UK). The PCR reactions were carried out in a 25 μl reaction mixture containing 2 μl cDNA, 1X Taq DNA polymerase buffer with MgCl_2_, 0.2 mM of each dNTP (MBI Fermentas, USA), 1.0 unit of Taq DNA polymerase (Bangalore Genie, India), and 10 pmol of appropriate primers for 28 cycles. The individual PCR amplified product was mixed with 6X loading dye (30% glycerol, 0.25% bromophenol blue and 0.25% xylene cyanol) and was separated by 2% agarose gel electrophoresis using 1XTAE buffer (40 mM Tris, 40 mM acetic acid and 1mM EDTA) as tank buffer containing ethidium bromide. The DNA bands were visualized in UV-Transilluminator and images of the gel were captured using digital camera. The photographic image was densitometrically scanned and quantified using Flurochem software, version 2.0 (Alpha Innotech, USA). The integrated density value (IDV) of the GluR2 cDNA band as obtained from above was divided by the IDV of the β-Actin and result was expressed as relative density value (RDV).

### Correlation of STZ-induced DM2- and CDRI-08-induced alterations in spatial memory with MDA level and expression of AMPA receptor GluR2 subunit

To analyze whether STZ-induced DM2- and CDRI-08–induced alteration in spatial memory are correlated oxidative stress due to lipid peroxidation, a correlation graph between spatial memory (escape latency) and MDA levels was plotted. A similar correlation graph was plotted between escape latency and levels of GluR2 subunit protein and mRNA levels in order to understand the relationship between STZ-induced DM2- and CDRI-08-induced alterations in spatial memory (escape latency) and expression of GluR2 gene at transcript and protein levels (relative density values measured in respect to β-Actin expression). Values of the correlation parameters have been shown in tabular form in S2 Table.

### Statistical analysis

All the experiments were repeated at least three times using a batch of 6–7 mice per experimental group. The individual data were expressed as mean ± SEM and densitometric values of the gels images or X-ray films were expressed as RDV for X-ray images of Western blotting and semi quantitative RT-PCR as bar diagram expressing mean ± SEM. Significance of the inter group data was analyzed by One-way ANOVA followed by Bonferroni multiple comparison post hoc tests using two-tailed *p* values using SPSS-16 software. The *p* values <0.05 was considered significant.

## Results

### STZ treatment leads to decline in body weight and CDRI-08 reverses it towards normal

**Fig 1:**
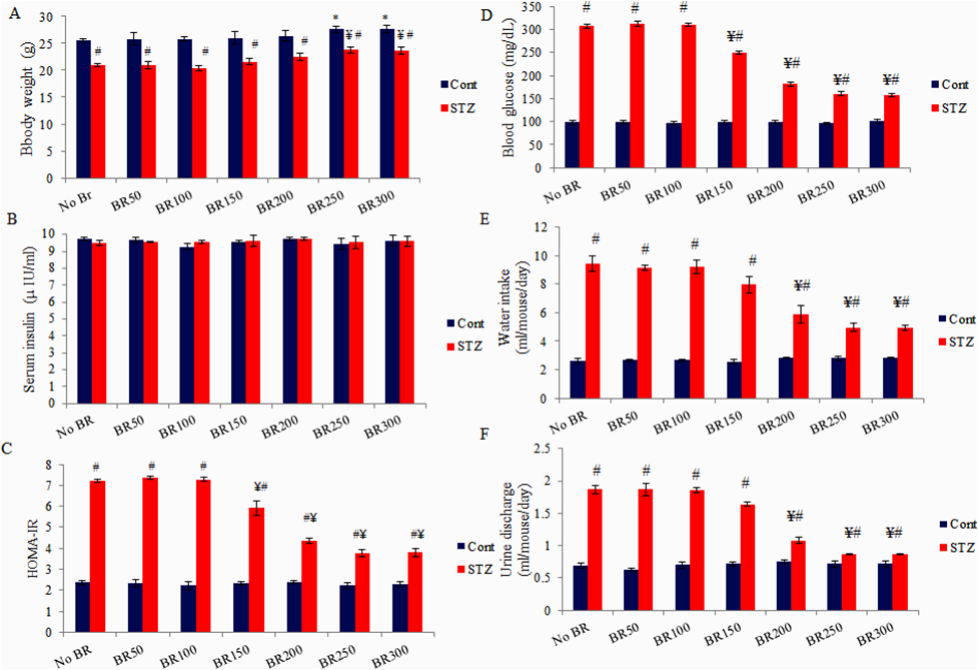
Effects of CDRI-08 on diabetic parameters in normal and CDRI-08 control and DM2 mice. A) Body weight (g/mouse); B) Serum insulin (μIU/ml); C) Insulin resistance (HOMA-IR); D) Blood glucose content (mg/1dL);E) Water consumption (ml/mouse/day) and F) Urine discharge (ml/mouse/day). Bar values represent mean ± SEM. Cont, Control; STZ, Streptozotocin treated; BR, CDRI-08 treated (figures on the x axis indicate CDRI-08 dose in mg/kg BW of mice; * denotes comparison within groups of control and different dose of CDRI-08 alone; ¥ denotes comparison within groups of DM2 and CDRI-08 treated DM2 mice; # denotes comparison between same dose of CDRI-08 in normal and DM2. *, # & ¥ denote *P*<0.05).Details of fasting serum insulin and glucose content and the corresponding HOMA-IR data are shown in S1 Table.

Our data on the effect of STZ reveals that STZ treatment leads to a significant decline in the body weight of STZ-treated mice compared to normal control mice. Oral treatment of CDRI-08 at dose between 50–200 mg/kg BW has no significant effects. However, treatment of DM2 mice with its higher dose (250- and 300 mg/kg BW) leads to a significant increase in the BW of DM2 mice near to the normal value. To study the per se effects ofCDRI-08, the STZ untreated mice were orally given different doses of CDRI-08 as was used in the treatment of STZ treated mice. Our data in this respect suggests that its dose up to 200 mg/kg BW has no significant effect on the body BW, however, when the control mice are treated with its higher dose 250- or 300 mg/kg BW, there is significant increase in their BW (**Fig 1A**).

### STZ-induced DM2 leads to increase in blood glucose content, water intake and urine output and CDRI-08 reverses them towards their normal values

Data obtained from our experiments on the effects of CDRI-08 on other diabetes mellitus parameters in mice are shown in **Fig 1**. Our data clearly indicates that streptozotocin treatment significantly increases the blood glucose levels compared to that in the control mice (*P*<0.05) i.e. it successfully produces a diabetic mice model, which can be used for further experiments. Our data further suggests that lower dose of CDRI-08 (50- or 100 mg/kg BW) does not affect the blood glucose content whereas its higher doses (150-, 200-, 250- and 300 mg/kg BW) lead to significant decline in the blood glucose content in general and above 200 mg/kg BW in particular also have pronounced antidiabetic effects (*P*<0.05). In order to study the per se effects of CDRI-08, it was observed that different doses of CDRI-08 alone have no significant effect on the blood glucose content (**Fig 1D**). To evaluate another condition of diabetes-induced conditions called polydipsia i.e. increase in water consumption (increased thirst) was measured as daily water intake by mice of all experimental groups. The results indicate that STZ treatment induces significant increase in the level of polydipsia in diabetic mice. Low dose CDRI-08, which does not affect the blood glucose content, does not also affect the level of polydipsia. However, the higher doses of CDRI-08 especially 250- or 300 mg/kg BW of CDRI-08 result in to significant decline in daily water consumption among diabetic mice near to normal. Also, it was observed that CDRI-08 alone, at any dose used for the treatment of STZ-mice, has no significant effect on the level of polydipsia (**Fig 1E**). In respect to assessing the diabetes-induced polyurea (increased urine discharge), it is observed that STZ-induced diabetic conditions leads to significant increase in the urine output (*p*<0.05). Whereas low doses of CDRI-08 were not able to reduce the urine output, the higher doses particularly above 200 mg/kg BW significantly reduced the level of polyurea near to normal value (*P*<0.05). CDRI-08 alone has no significant effect on the level of polyurea (**Fig 1F**).

### CDRI-08 reverses insulin resistance in STZ- treated DM2 mice to normal in dose-dependent manner

Data obtained from the ELISA of insulin reveals that STZ treatment does not affect the serum insulin level compared to that in the normal control mice. The data signifies that the STZ-treated pups which grew up to adult age did not suffer from the adverse effects of the STZ that would have otherwise decreased the serum insulin level in adult age. It indicates that the resulting decline in the blood sugar level in STZ-treated mice might be due to failure of the insulin targeting leading to alterations in the glucose metabolism. Data on the effects of CDRI-08 alone reveals that it does not affect the pancreatic secretion of insulin and its level in the blood (**Fig 1B**). In regard to assessing the insulin targeting function, we measured the insulin resistance values in experimental and the corresponding control mice. Our HOMA-IR data reveal that STZ treatment significantly increases the insulin resistance compared to the control mice that were treated with citrate buffer (vehicle) (p<0.05). This clearly indicates that the STZ-treated mice developed DM2. Our data further indicate that the CDRI-08 doses up to 100 mg/kg BW, when given to STZ-DM2 mice, do not alter the insulin resistance values compared to STZ treated mice, however, the higher doses such as 150- and 200 mg/kg BW leads to a gradual decline in the resistance values compared to its lower doses. Further higher CDRI-08 doses i.e. 250 and 300 mg/kg BW lead to an additional decline in the resistance close to its value in the normal control mice (*P*<0.05) (**Fig 1C, S1 Table**). Further, it is also observed that all the doses of CDRI-08 alone do not have any significant effect on the insulin resistance values compared to the normal mice but they possess resistance lowering potentials in dose dependent manner when given to DM2 mice.

### CDRI-08 recovers spatial memory impairment due to STZ-induced DM2

**Fig 2:**
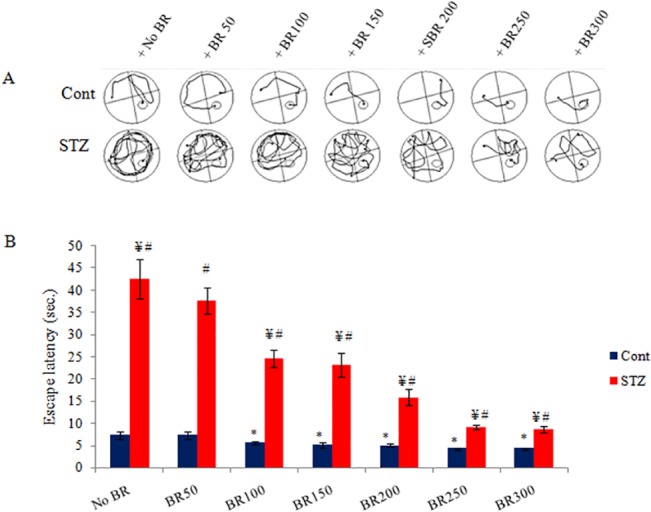
Effects of CDRI-08 on the spatial memory in normal and CDRI-08 control and DM2 mice. A) Track path of mice in Morris-water- maze pool; B) Escape latency (Sec). Bar values represent mean ± SEM. Cont, Control; STZ, Streptozotocin-treated; BR, CDRI-08 treated (figures indicate CDRI-08 dose in mg/kg BW of mice; * denotes comparison within groups of control and different dose of CDRI-08 alone. ¥ denotes comparison within groups of DM2 and CDRI-08 treated DM2 mice; # denotes comparison between same dose of CDRI-08 in normal and DM2. *, # & ¥ denote *P*<0.05).

Our data from the Morris-water-maze test for assessing the level of spatial memory in mice reveals that the STZ-treated diabetic mice uses long escape latency and covers long distance for searching the hidden platform in the water pool compared to normal control (non-diabetic) mice. That means, the STZ-induced diabetic mice exhibit significantly decreased spatial memory in comparison to the normal level of spatial memory exhibited by non-diabetic mice (~8-fold decrease, *p*<0.05). Thus our data confirms that the conditions of diabetes mellitus lead to spatial memory loss in diabetic mice. To study the beneficial effects of the CDRI-08, its various doses were orally administered to STZ-treated diabetic mice for 15 days. The results suggest that all doses of CDRI-08 were able to significantly decrease the escape latency time and the distance travelled for exploring the hidden platform but effects of higher doses had remarkably significant effects on recouping the memory loss back to normal (*p*<0.05) and this recovery of diabetes-induced memory loss by CDRI-08 is largely related to its high dose-dependent antidiabetic effects on the brain function (**Fig 2B**). In order to assess whether, the above procognitive effect of CDRI-08 is due its own neuroprotective effects, a separate set of non-STZ treated mice (CDRI-08 control) were orally given similar different dose of CDRI-08 alone as was given to STZ-treated mice and all the mice were individually subjected to Morris-water-maze test. Our spatial memory data reveals that there is significant and gradual decline in the escape latency due to treatment of non-STZ treated normal control mice with CDRI-08 above 50 mg/kg BW doses. Treatment of mice with 50 mg/kg BW dose did not show any significant change in the escape latency (**Fig 2A**). The data clearly suggests that the CDRI-08 alone also has memory enhancing effects but extent of effects of its corresponding doses is more pronounced when they are administered to STZ-treated DM2 mice.

### CDRI-08 decreases the level of lipid peroxidation in the hippocampus of STZ-induced DM2 mice

**Fig 3:**
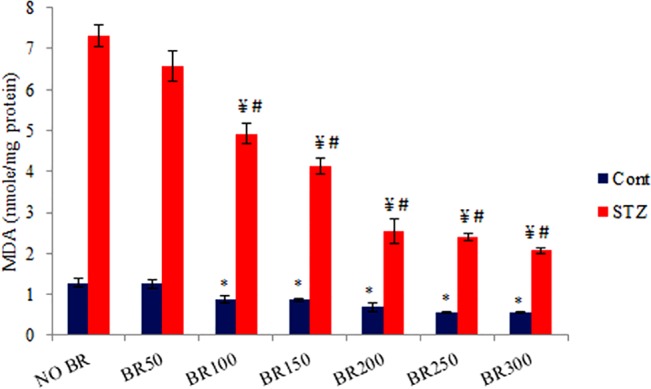
Effects of CDRI-08 on the level of MDA in hippocampus of normal and CDRI-08control and DM2 mice. Bar values represent mean ± SEM. Cont., Control; STZ, Streptozotocin treated; BR, CDRI-08 treated (figures indicate CDRI-08 dose in mg/kg BW of mice; Cont, Control; STZ, Streptozotocin treated; BE, CDRI-08 treated (figures indicate dose in mg/kg BW of mice; * denotes comparison within groups of control and different dose of CDRI-08 alone; ¥ denotes comparison within groups of DM2 and CDRI-08 treated DM2 mice; # denotes comparison between same dose of CDRI-08 in normal and DM2. *, # & ¥ denote *P*<0.05).

To evaluate the impacts of STZ-induced DM II, level of lipid peroxidation i.e. malonaldehyde (MDA) was determined in the hippocampus of mice of all experimental groups. Our data reveals that STZ treatment leads to drastic increase in the level of lipid peroxidation (~6-fold increase) when compared with the same in normal control mice (*p*<0.05). CDRI-08 treatment up to 150 mg/kg BW significantly reduces the level of lipid peroxidation, however, its treatment with higher doses (200 mg/kg BW onward) results into further significant decline in its level. This further confirms that DM2-induced impairment in the spatial memory is due to increased oxidative stress. Further, the CDRI-08 has restorative action in reversing the spatial memory loss towards normal and it is associated with its anti oxidative stress function. Further, when different doses of CDRI-08 alone were given to non-STZ-treated control mice, it showed a pattern of dose-dependent decline in MDA content of hippocampus; however, the effects were at low levels. Extent of effects of CDRI-08 was significantly more prominent at 200 mg/kg BW dose and above in STZ-treated DM2 mice compared to its effect alone or in DM2 mice with dose 150 mg/kg BW or less (**Fig 3**).

### CDRI-08 up regulates expression of the AMPA receptor GluR2 subunit in the hippocampus of STZ-induced DM2 mice

**Fig 4:**
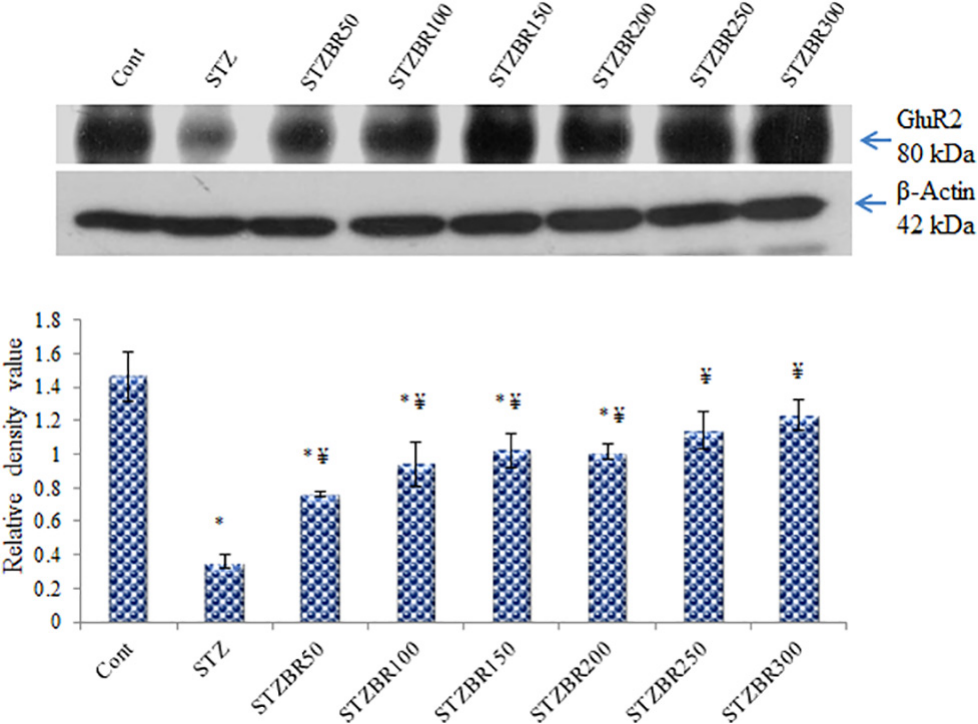
Western blot analysis of CDRI-08 effects on the expression of GluR2 subunit protein in the hippocampus of normal control and DM2 mice. Bar values represent mean RDV ± SEM. Cont, Control; STZ, Streptozotocin treated; BR, CDRI-08 treated (figures indicate CDRI-08 dose in mg/kg BW of mice; * denotes comparison between experimental groups and control; ¥ denotes comparison between STZ- treated (diabetic) mice. *, # & ¥ denote *P*<0.05).

**Fig 5:**
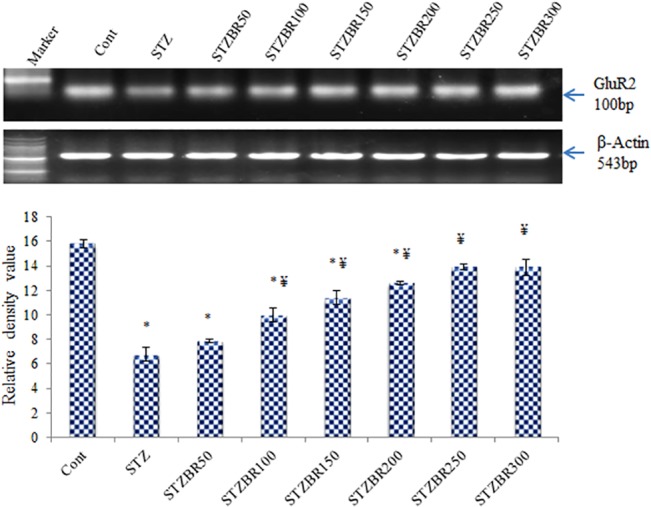
Semi quantitative RT-PCR analysis of effects of CDRI-08 on expression of GluR2 subunit transcript in hippocampus of the normal control and DM2 mice. Bar values represent mean RDV ± SEM. Cont, Control; STZ, Streptozotocin treated; BR, CDRI-08 treated (figures indicate CDRI-08 dose in mg/kg BW of mice; * denotes comparison between experimental groups and control; ¥ denotes comparison between STZ- treated (diabetic) mice. *, # & ¥ denote *P*<0.05).

Our Western blotting data reveals that the STZ-induced DM2 leads to a significant decline in the expression of AMPA receptor GluR2 subunit protein in the hippocampus of mice (~ 6-fold, *p*<0.05). Data also suggests that CDRI-08 significantly up regulates the expression of GluR2 subunit at lower doses (50- and 100 mg/kg BW) whereas the dose range of 150–200 mg/kg BW has no significant effects. However, the CDRI-08 increases the expression of GluR2 subunit significantly at higher dose i.e. 250- and 300 mg/kg BW (*p*<0.05) (**Fig 4**). The semi quantitative RT-PCR data reveals the similar patterns of effects of the STZ-induced DM2. Further, the expression of GluR2 transcript is gradually increased with increase in the dose of CDRI-08 up to 250 mg/kg BW, however, it has no significant effect at 300 mg/kg BW (**Fig 5**). Decline in the expression of GluR2 subunit gene in the hippocampus is correlated with decline in the spatial memory and CDRI-08-inducedup regulation is associated with its recovery in the STZ-induced DM2 mice. The results are suggestive of the therapeutic potentials of the CDRI-08.

**Fig 6:**
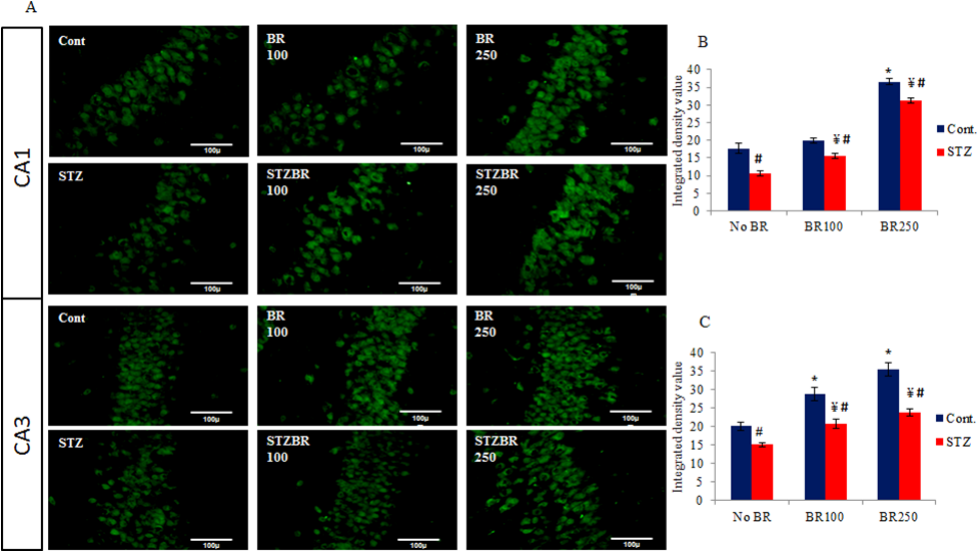
Immunofluorescence analysis of the effects of CDRI-08 on expression of the GluR2 subunit protein in hippocampus area CA1 and CA3 of normal and DM2 mice. A) Photomicrograph showing immunoflorescence signal of GluR2 subunit in CA1 and CA3 regions of hippocampus, Bar diagram showing FITC signal intensity in B) CA1 and C) CA3 hippocampal regions, separately. Bar values represent mean ± SEM. Cont., Control; STZ, STZ-treated; BR, CDRI-08 treated (figures indicate CDRI-08 dose in mg/kg BW of mice; Cont, Control; STZ, STZ-treated; BR, CDRI-08 treated (figures indicate CDRI-08 dose in mg/kg BW of mice; * denotes comparison within groups of control and different dose of CDRI-08 alone; ¥ denotes comparison within groups of DM2 and CDRI-08 treated DM2 mice; # denotes comparison between same dose of CDRI-08 in normal and DM2*, # & ¥ denote *P*<0.05), Scale bar = 100μ.

To study the per se effects of CDRI-08 alone, its various doses were administered orally to normal and STZ-treated DM2 mice separately. The effects on the expression of GluR2 subunit at protein level were analyzed by immunofluorescence microscopy in order to ascertain the immunoblot data. For this purpose, unlike those in the immunoblot analysis, two representative doses- one among the lower doses i.e. 100 mg/kg BW and the other among the higher doses i.e. 250 mg/kg BW of CDRI-08 were chosen. They were orally given to separate batches of normal control and STZ-treated mice. The immunofluorescence signals were separately analyzed in CA1 and CA3areas of the hippocampus. Our immunofluorescence data reveals that STZ treatment leads to significant decline in the level of GluR2 subunit in both CA1 and CA3 areas of the hippocampus compared to that in the normal control mice. Treatment of STZ-DM2 mice with100- or 250 mg/kg BW doses of the CDRI-08 significantly increases the expression of GluR2 subunit towards their normal control values in both CA1 and CA3 areas. Treatment of the STZ-untreated mice (normal control) with 100 mg/kg BW of CDRI-08 alone does not have any significant effect on the level of GluR2 subunit in the CA1 area whereas its higher dose (250 mg/kg BW) has significant effect. However, both the doses of CDRI -08 up regulate the expression of the GluR2 subunit in CA3 area indicating the hippocampal area dependent regulatory role of CDRI-08 per se in the expression of GluR2 subunit. Also, our data suggests that STZ treatment reduces the extent of reversal effects of CDRI-08 on the expression of GluR2 subunit (**Fig 6**).

### CDRI-08-induced alteration in spatial memory is correlated with reversal of STZ-induced effects on MDA level and AMPA receptor GluR2 subunit expression

**Fig 7:**
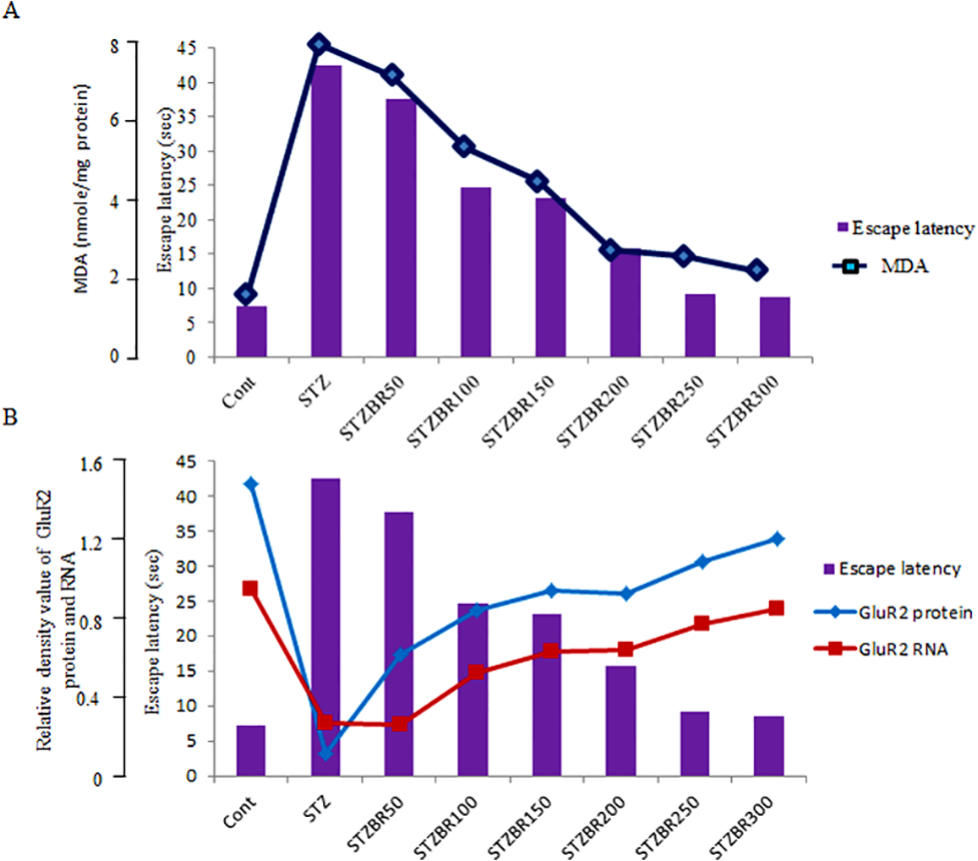
Correlation/association analysis between CDRI-08 dose-dependent alterations in spatial memory performance (escape latency) and oxidative stress (MDA level) in normal control and in DM2 mice (A) and GluR2 subunit protein and transcript levels in normal control and DM2 mice (B). Association analysis related values are shown in S2 Table.

Our correlation data suggests that the STZ treatment-caused decline in spatial memory is correlated with is associated with increase in lipid peroxidation, the mark of the oxidative stress.CDRI-08 (all doses)-dependent gradual enhancement of spatial memory in STZ-induced DM2 mice is correlated with gradual decline in lipid peroxidation (**Fig 7A, S2 Table**). Further, the correlation data between spatial memory and the AMPA receptor GluR2 subunit gene expression suggest that the spatial memory decline due to STZ treatment is correlated with lower expression levels of both GluR2 protein and transcript in the hippocampus in comparison to the normal control mice. Data also suggests that as the CDRI-08 dose increases, the escape latency decreases i.e. the spatial memory level increases and the RDV of GluR2 i.e. level of both GluR2 subunit protein and transcript, respectively also increases. Thus it clearly indicates that CDRI-08-induced spatial memory enhancement is correlated with gradual increase in the expression of GluR2 subunit in dose-dependent manner (**Fig 7B, S2 Table**).

## Discussion

We have studied effects of diabetes mellitus type 2 (DM2) on the spatial memory in mice and its association with the oxidative stress and expression of AMPA receptor GluR2 subunit gene in the hippocampus as this is the major site in the brain for consolidation and storage of the long term memory. In order to study above, we developed a type 2 DM (DM2) mouse model by intraperitoneal injection of a selected dose of streptozotocin established from our pilot experiment and validated the model by examining the diabetic parameters like blood glucose content (hyperglycemia), level of polydipsia (excess need of water intake) and polyurea (excessive urine discharge). Since, the DM2 is also associated with change in body weight and alterations in serum insulin level or its sensitivity; we also measured the body weight, serum insulin level and insulin resistance in different controls as well as experimental mice. The DM2 mice were further used for analyzing alterations in the spatial memory and its association with oxidative stress at the level of lipid peroxidation and GluR2 submit gene expression. In order to investigate the possible mechanisms of CDRI-08effects on DM2-induced memory impairment, and since the optimum dose of the CDRI-08 is not well established, we studied effects of its different doses on alterations in spatial memory and its association with lipid peroxidation and GluR2 subunit expression in the hippocampus of the STZ-induced DM2 mice and compared the data with those in the normal and CDRI-08 treated control mice.

Numerous methods have been followed by diabetes researchers for developing models of type 2 DM by injecting STZ in mice or the rats of different strains as per their respective experimental strategies [38,49–52]. We have followed a particular one as has been described in the materials and methods [38]. Despite the DM2 mouse model chosen by us is a suitable model, we further carried out a pilot experiment for developing type 2 diabetic model by injecting different doses of STZ to Swiss strain mice pups and measured the serum glucose level in each case, checked the mortality rate in group of STZ-treated mice and compared the data with normal control mice. We observed that 50-or 75 mg STZ/kg BW was not able to develop DM2 whereas 100 mg STZ/kg BW and above could develop diabetes successfully, however, the STZ dose 125 mg/kg BW and above caused mortality in mice. Treatment of mice with 100 mg/kg BW of STZ did not result into death of mice. Therefore, we chose a dose of 100 mg/kg BW of STZ for developing DM2 mouse model and validated the model for salient features of diabetes. Our validation data reveal that streptozotocin-treated mice developed the characteristic features of diabetes mellitus such as increased blood glucose level (hyperglycaemia), increased water consumption (polydipsia) and excessive urine discharge (polyurea).It was also observed that STZ mice exhibited significant decline in body weight compared to the normal control mice, and the treatment of STZ mice with CDRI-08 doses up to 200 mg/kg BW had no remarkable effects on the body weight, however, the dose above it i.e. 250 or 300 mg/kg BW caused slight increase in the body weight in CDRI-08 control as well as DM2 mice but the extent of the body weight increase was lower in DM2 mice. To know whether these changes in DM2 mice are associated with serum insulin content or insulin resistance, we measured the serum insulin level and insulin resistance value (HOMA-IR). We observed that alterations in various diabetic parameters were not associated with alterations in insulin level rather it was associated with alterations in insulin resistance value in CDRI-08 dose-dependent manner. The current observation is important as insulin signaling plays critical role in the regulation of food intake, body weight, reproduction, learning and memory [53]. Disruption of insulin signaling is known to make neurons vulnerable to metabolic stress and accelerate neuronal dysfunction leading to decreased cognitive ability and development of the symptoms of dementia [54]. Poor cognitive performance in diabetes has been associated with increase in insulin resistance and decline in cerebrospinal fluid (CSF) insulin level [55]. Our current data on the loss of body weight in DM2 mice and increased blood glucose level due to increased insulin resistance and CDRI-08 induced slight gain in body weight due to decline in insulin resistance corroborates with the earlier reports by Das 1999 [56,57]. As is observed in our study, level of insulin does not decrease due to 12 week STZ treatment and it is maintained at the level near to normal control mice and CDRI-08 treatment does not have any effect on its. This finding on the unaltered level of insulin in post 12 week STZ treated DM2 mice compared to the normal may be because of histogenesis of pancreatic duct cells after the STZ-induced complete destruction of β cells of islets of Langerhans in the pancreas in mice pups in order to compensate the higher insulin demand of the body later in adult age [58]. Thus the question arises on the relation between unaltered insulin level and decreased blood glucose content in DM2 mice after CDRI-08 treatment. In this regard, our results suggest that CDRI-08 which, in its lower dose range, has no effects on the serum level of insulin, also has no effects on insulin resistance values, however, the its higher doses decrease the insulin resistance as evident from our HOMA-IR data, which can be correlated with its anti hyperglycaemic effects.

One of the most important purposes of the current study was to assess whether DM2 has effects on learning and memory. We, therefore, investigated whether the STZ-treated mice suffer from spatial memory loss/impairment. The Morris-water-maze track record data, as analyzed by Any Maze Software, clearly suggest that STZ-induced diabetes mellitus type 2 (DM2) leads to spatial memory loss. Similar results on the adverse effects of STZ on memory have also been reported by researchers in rodent models [59,60]. This decline in spatial memory has been correlated with initial decline in the level of insulin in the blood due to toxic effects of streptozotocin on the β cell of the pancreatic islets of Langerhans and reduced insulin signalling in the hippocampus or the other memory related brain regions as they have been reported to have insulin receptor [61,62] or increase in the insulin resistance value [10]. Recovery of the memory loss by higher doses of CDRI-08 can be correlated with decline in insulin resistance which could lead to decline in the serum glucose towards normal control value as evident from our data whereas pro cognitive effects of its lower doses might be associated with decline in oxidative stress as observed by us. In addition to above and also as observed in our current study, the DM-induced factors like activation of polyol-sorbitol pathways [13] leading to increase in the oxidative stress by accumulation of reactive oxygen species (ROS) due to decline in activities of antioxidative stress enzymes and shift of their balance from antioxidative stress to oxidative stress in neurons may result into possible reduction in the expression of LTP and increase in LTD and thereby impairment of memory in DM2 mice [63]. Our data confirms that STZ-induced DM2 increases oxidative stress by increasing the level of MDA due to increased lipid peroxidation in the hippocampus. This increase in the oxidative stress might be attributed to decline in the activities of anti oxidative stress enzymes like superoxide dismutase (SOD), catalase (CAT) and glutathione peroxidase (GPx) that could lead to accumulation of reactive oxygen species (ROS)in the hippocampus. In our laboratory, it has been shown that STZ-treated DM2 mice possess decreased activities of anti oxidative stress enzymes- SOD and CAT in the hippocampus (unpublished data). This decline in the anti oxidative stress system or increase in the oxidative stress is known to decrease the cognitive function of the brain by disrupting synaptic plasticity [64]. Further, due to high blood glucose and oxidative stress, blood brain barrier has been reported to become more leaky to harmful substances which, in turn, may exaggerate the effects of oxidative stress in the brain [65] and alter its structure and function [66] due possibly to apoptosis of neurons [67]. This may altogether severely compromise the function of various memory related regions in the brain including hippocampus [68].

To ensure the DM2-induced increase in the oxidative stress leading to decline in learning and memory or cognition, we thought to investigate the expression of one of the synaptic plasticity related genes called ionotropic glutamate receptor i.e. AMPA receptor gene. Our data supported that as the level of oxidative stress increased, it resulted into significant decline in expression of the AMPA receptor GluR2 subunit gene in the hippocampus of the STZ-treated DM2 mice, which might be associated with the abnormal development of synaptic plasticity during learning and memory [69]. Also, this is likely to lead to decrease in the activity of another ionotropic glutamate receptor called NMDA receptor on the post synaptic density which might in turn lead to poor Ca^2+^signallingin the post synaptic neurons and hence the negative alterations in synaptic plasticity and decline in memory [23]. However, diabetes mellitus has also been reported to enhance AMPA- and NMDA receptors activity leading to excitoneurotoxicity in various brain regions and it has been associated with alterations in cognitive ability of animals and patients [22].

For understanding whether the CDRI-08 has effects on learning, memory and cognition, we orally administered its different doses to the STZ-treated DM2 mice and subjected them to Morris-water-maze test. The data on the software based analysis of spatial memory suggests that the CDRI-08 has pro cognitive or neuroprotective effects i.e. it recovers the spatial memory loss in the STZ-DM2 mice. It also reveals that CDRI-08 especially above 150 mg/kg BW leads to reversal of the increased diabetes related markers such as blood glucose content, water consumption and urine discharge towards normal in DM2mice. CDRI-08-dependent decline in the blood glucose content towards normal value was not found to be associated with increase in the insulin level, however, it was found to be associated with decline in insulin resistance especially by its dose between 150–300 mg/kg BW. This was altogether associated with a significant shift of the conditions of oxidative stress to antioxidative stress side by down regulating the level of MDA i.e. decline in lipid peroxidation (the index of oxidative stress) in the hippocampus. The anti diabetic potential of the higher dose of CDRI-08 (i.e. 150–300 mg/kg BW) by increasing the insulin sensitivity is a novel observation in our current study, which is consistent with data reported earlier on the anti diabetic role of *Bacopa monnieri* whole extract [70].This role of CDRI-08 corresponds to known antioxidant potential of *Bacopa monnieri* [71] which underlies the recovery of cognitive deficiency as observed by us based on Morris-water-maze test data [72]. We have also shown the anti oxidant potential of CDRI-08 in STZ treated DM2 mice based on increase in the activities of SOD and CAT in the hippocampus (unpublished data). However, the precise molecular mechanisms underlying pro cognitive role of CDRI-08 is not well known. Our study provided the evidence of the molecular basis of role of CDRI-08 in the recovery of memory loss and thereby recovery of the altered synaptic plasticity by up regulating the expression of AMPA receptor GluR2subunit in the hippocampus of DM2 mice that plays crucial role in learning, memory and cognition. All the doses of CDRI-08 especially above 100 mg/kg BW were able to up regulate the expression of GluR2 subunit gene in the hippocampus of the diabetic mice. Effects of CDRI-08 in up regulation of GluN2B subunit of the NMDA glutamate receptor and *Fmr-1* gene expression in scopolamine-induced amnesia and cobalt chloride-induced hypoxic mice, respectively have recently been reported by us [73,74]. As per recent evidence, *Bacopa monnieri* alcoholic extract has been reported to possesses neuroprotective action by reversing the memory impairment due to pilocarpine treatment in rats by up regulating the expression of the NMDA glutamate receptor GluN1 subunit in the hippocampus [75] and activity of the NMDA receptor in the cerebral cortex [76]. Also, it is likely that the CDRI-08modulates various neurotransmitters such as acetylcholine (ACh), serotonin, GABA, glutamate and dopamine in different brain regions that play role in enhancement of the cognitive functions [37,77]. A similar report has been published on effects of the curcumin, a known antioxidant, on the recovery of memory loss in rats by lowering the excitotoxicity by modulating the role of AMPA and NMDA glutamate receptors and thereby the synaptic plasticity in STZ-induced diabetic rats [22]. The neuroprotective role of CDRI-08, as observed in our study, is consistent with the role of Bacosides A and B content [72,78] in the recovery of conditions of several neuronal disorders such as anxiety, depression and epilepsy [31–33]. From our data, it is also revealed that CDRI-08 alone is not effective on many of the parameters of type 2 diabetes excepting improving memory performance to some extent compared to control mice, but when it is administered after the development of DM2, its role become paramount at all scales as discussed above in dose-dependent manner. Our data also indicates that the CDRI-08 dose between 250–300 mg/kg BW possesses anti diabetic property. However, when it is translated into human diabetic patients, its final effective dose will be in terms of grams/day which looks a bit impractical. But, it is only a preclinical observation on its anti diabetic function in addition to its role in enhancement of memory performances in diabetic mice, however, the possible side effects of such higher CDRI-08 doses cannot be ruled out. We have characterized basis of memory enhancing function of CDRI-08 which is only a fraction of the whole extract of *Bacopa monnieri* rich in bacosides A and B 9 (57.5%). Therefore, it is likely that when the active principles i.e. bacosides A and B are separated from other ingredients of the whole extract and which might have been active at low dose being together, may lose its efficacy and thus may require high dose to produce the similar effects as brought by the *Bacopa monnieri* total extract. This could be one of the possible reasons for a need of higher dose range of CDRI-08 in correcting the memory impairments along with its antidiabetic effects by increasing the targeting function of the insulin in glucose metabolism.

Recent studies have shown that streptozotocin changes the property of the membrane by affecting its associated fatty acids and their transporter [79], altering the associated proteins that maintain its electrical properties [22], causing misfolding of cellular proteins [80], affecting vesicular membrane associated proteins SNAP23, syntaxin-4 and VAMP-2, SNARE, and glucose transporter (GLUT-4) [81], which, in turn, induces diabetes or diabetes induced cellular or organ dysfunction. Therefore, it is likely that CDRI-08, which shows anti oxidative stress and antidiabetes associated procognitive properties, as observed in the current study, may alter the STZ-induced abnormalities in the membrane or cellular function. Recent studies have shown that *Bacopa monnieri* extract modulates release of neurotransmitters-serotonin, dopamine, acetylcholine, and γ-amino butyric acid in executing its pharmacological effects to improve the neural plasticity [82]. It has also been that the *Bacopa* extract alters the GABA receptor activity in the cortex of epileptic rats [83] and increases the level of NMDAR1 on the post synaptic membrane in various memory associated regions of the brain in rat model of schizophrenia [84]. Thus our study indicates that CDRI-08 may overcome the STZ-induced alterations in the membrane by either altering the structure/properties or number of the AMPA recptors and thus the structure/properties of the post synaptic membrane of the glutametrgic synapse in the hippocampus and restore the spatial memory by increasing insulin sensitivity and thereby decreasing the oxidative stress.

Our findings provide the biochemical and molecular basis of the role of CDRI-08 in recovery of the DM2-induced memory loss in mice by modulating the expression of the AMPA type glutamate receptor by way of reversing the increased blood glucose level to normal by decreasing the insulin resistance and thereby decreasing the oxidative stress in dose-dependent manner. Thus CDRI-08 has a neuroprotective function in respect to cognitive loss due to diabetes mellitus as has been observed in earlier studies also. In addition to its neuroprotective function, our study also suggests that CDRI-08 has antidiabetic potential. Therefore, the CDRI-08 has therapeutic implications. So far as its role in treating diabetes mellitus is concerned, it may safely be used as a complementary drug with known antidiabetic drugs; however, it needs a thorough study. CDRI-08 may have its beneficial effects by insulin signalling dependent synaptogenesis, expression and activities of NMDA receptor and AMPA receptor, their trafficking proteins, dendritic spine morphology and synaptic plasticity which may be correlated with enhancement of memory in DM2 animals/patients. They need to be adequately and carefully examined using electrophysiological parameters which might enhance the scope of study on the effects of CDRI-08 on the memory altered due to DM2.

## Supporting Information

S1 Fig Effects of different doses of STZ on blood glucose content. 50, 75, 100, 125 and 150 mg/kg BW were administrated (ip) in 0 day male pups and blood glucose content was analyzed after 4-, 6-, 8–12 and 16 week and results were expressed as bar diagram. Number on each bar represents the number of deaths of mice. * denotes comparison within groups of control of 4 week and different dose of STZ; # denotes comparison within groups of control of 8 week and different dose of STZ; # denotes comparison within groups of control of 12 week and different dose of STZ; μ denotes comparison within groups of control of 16 week and different dose of STZ; *, #, μ & ¥ denote P<0.05). (TIF)
(TIF)

S1 Table Values of fasting serum insulin level, fasting blood glucose content and corresponding HOMA-IR values obtained from mice of various experimental groups. STZ, streptozotocin treated mice; STZ+BR, Streptozotocin-treated mice treated with CDRI-08; Values indicate various doses of CDRI-08 (mg/kg BW of mice).
(DOCX)

S2 Table Correlation study between spatial memory (escape latency, which is denoted as ‘x’) in respect to MDA level, GluR2 subunit protein and GluR2 subunit mRNA levels in response to treatment of various doses of CDRI-08 treated STZ-DM2 mice. (**Table**)
(DOCX)
