# Supplementary figures and images for: Alterations in Hippocampal Oxidative Stress, Expression of AMPA Receptor GluR2 Subunit and Associated Spatial Memory Loss by Bacopa monnieri Extract (CDRI-08) in Streptozotocin-Induced Diabetes Mellitus Type 2 Mice
Source: PLoS One. 2015 Jul 10;10(7):e0131862. doi: 10.1371/journal.pone.0131862 (PMC4498885; doi:10.1371/journal.pone.0131862)

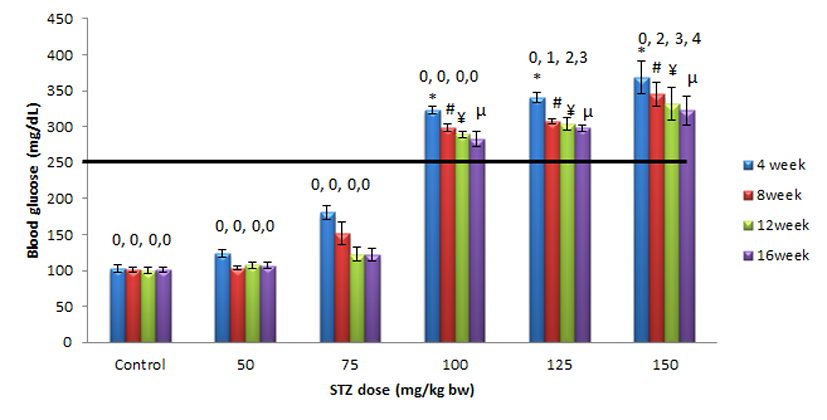

Supplement: S1 Fig — 50, 75, 100, 125 and 150 mg/kg BW were administrated (ip) in 0 day male pups and blood glucose content was analyzed after 4-, 6-, 8–12 and 16 week and results were expressed as bar diagram. Number on each bar represents the number of deaths of mice. * denotes comparison within groups of control of 4 week and different dose of STZ; # denotes comparison within groups of control of 8 week and different dose of STZ; # denotes comparison within groups of control of 12 week and different dose of STZ; μ denotes comparison within groups of control of 16 week and different dose of STZ; *, #, μ & ¥ denote P<0.05). (TIF) (TIF) [file pone.0131862.s001.tif]
