# Supplementary material for: Alterations in Hippocampal Oxidative Stress, Expression of AMPA Receptor GluR2 Subunit and Associated Spatial Memory Loss by Bacopa monnieri Extract (CDRI-08) in Streptozotocin-Induced Diabetes Mellitus Type 2 Mice
Source: PLoS One. 2015 Jul 10;10(7):e0131862. doi: 10.1371/journal.pone.0131862 (PMC4498885; doi:10.1371/journal.pone.0131862)
Supplement: S1 Table — STZ, streptozotocin treated mice; STZ+BR, Streptozotocin-treated mice treated with CDRI-08; Values indicate various doses of CDRI-08 (mg/kg BW of mice). (DOCX) [file pone.0131862.s002.docx]

**ESM 2**: Values of fasting serum insulin level and blood glucose content and corresponding HOMA-IR. STZ, streptozotocin treated mice; STZ+BR, Streptozotocin-treated mice treated with CDRI-08; Values shown below as BR50, BR100, etc. indicate various doses of CDRI-08 (mg/kg BW of mice.

| Exp. Set | Fasting serum insulin level (µIU/ml) | Fasting serum glucose content (mg/dL) | HOMA-IR value |
| --- | --- | --- | --- |
| Normal Control | 9.73 | 98.50 | 2.37 |
| STZ (without CDRI-08) | 9.50 | 306.83 | 7.20 |
| BR50 Control | 9.67 | 98.67 | 2.36 |
| STZ+BR50 | 9.53 | 312.65 | 7.36 |
| BR100 Control | 9.27 | 97.17 | 2.22 |
| STZ+BR100 | 9.53 | 309.50 | 7.29 |
| BR150 Control | 9.53 | 99.83 | 2.35 |
| STZ+BR150 | 9.60 | 249.50 | 5.91 |
| BR200 Control | 9.73 | 99.00 | 2.38 |
| STZ+BR200 | 9.73 | 181.17 | 4.35 |
| BR250 Control | 9.43 | 95.83 | 2.23 |
| STZ+BR250 | 9.53 | 160.50 | 3.78 |
| BR300 Control | 9.63 | 95.83 | 2.28 |
| STZ+BR300 | 9.60 | 160.50 | 3.80 |
