# Supplementary material for: Alterations in Hippocampal Oxidative Stress, Expression of AMPA Receptor GluR2 Subunit and Associated Spatial Memory Loss by Bacopa monnieri Extract (CDRI-08) in Streptozotocin-Induced Diabetes Mellitus Type 2 Mice
Source: PLoS One. 2015 Jul 10;10(7):e0131862. doi: 10.1371/journal.pone.0131862 (PMC4498885; doi:10.1371/journal.pone.0131862)
Supplement: S2 Table — (Table) (DOCX) [file pone.0131862.s003.docx]

**ESM 3:** Correlation study between spatial memory performance (escape latency, denoted as ‘x’) in respect to MDA, GluR2 subunit protein and GluR2 subunit mRNA levels in response to treatment of various doses of CDRI-08 treated DM2 mice..

| Correlation between | *y* values | Correlation coefficient (R^2^) |
| --- | --- | --- |
| MDA and Escape latency | 0.9115*x* + 8.9883 | 0.9757 |
| GluR2 Protein and Escape latency | -0.649*x* + 113.89 | 0.987 |
| GluR2 Transcript and Escape latency | -0.7023*x* + 113.15 | 0.8663 |
